# Attention-Deficit/Hyperactivity Disorder in Relation to Addictive Behaviors: A Moderated-Mediation Analysis of Personality-Risk Factors and Sex

**DOI:** 10.3389/fpsyt.2015.00047

**Published:** 2015-04-20

**Authors:** Caroline Davis, Alina Cohen, Mark Davids, Alex Rabindranath

**Affiliations:** ^1^Kinesiology and Health Sciences, York University, Toronto, ON, Canada

**Keywords:** attention deficit/hyperactivity disorder, addictive behaviors, personality, sex

## Abstract

**Introduction:**

Research has shown that those with attention-deficit/hyperactivity disorder (ADHD) have an increased risk for addiction disorders like alcoholism and substance abuse. What is less clear is the mechanism(s) whereby ADHD gives rise to increased engagement in addictive behaviors, and whether there are sex differences in the ADHD-addiction propensity. Both ADHD and addictions have also been associated with personality traits such as impulsivity, reward seeking, anxiousness, and negative affect. In this study, we tested a moderator-mediation model, which predicted that both sex and ADHD-symptom status would make independent contributions to the variance in personality risk and in addictive behaviors, with males, and those with diagnosed ADHD, scoring higher on both dependent variables. Our model also predicted that the effect of sex and ADHD-symptom status on addictive behaviors would be via the mediating or intervening influence of personality-risk factors.

**Methods:**

A community-based sample of young men and women took part in the study. Among these individuals, 46 had received a lifetime diagnosis of ADHD. The non-diagnosed participants were dichotomized into a high-ADHD-symptom group (*n* = 83) and a low-symptom group (*n* = 84).

**Results:**

We found that a high-risk personality profile may, in part, account for the relationship between ADHD symptomatology and the use/abuse of a broad range of addictive behaviors. However, we found no sex differences in personality risk for addiction or in the use of addictive behaviors; nor did sex moderate the relationships we assessed.

**Conclusion:**

While ADHD status showed a strong relationship with both dependent variables in the model, we found no difference between those who had been diagnosed with ADHD and treated with stimulants, and their high-symptom non-diagnosed/non-treated counterparts. These results add support to claims that the treatment of ADHD with stimulant medication neither protects nor fosters the risk for substance abuse disorders.

## Introduction

Attention-deficit/hyperactivity disorder (ADHD) is a highly heritable (≈70%) neuropsychiatric disorder with typical onset in childhood (1). Furthermore, in a substantial proportion (≈75%) of cases, ADHD symptoms do not remit in childhood/adolescence and continue into adulthood (2). It appears, however, that genetic factors account for a lower heritability in adults with ADHD (≈30–40%) than in children with this disorder (3, 4). The psychosocial and behavioral impairments that characterize ADHD are associated with a number of deleterious outcomes. Perhaps most notably is the increased risk of substance use and abuse – evidence, which derives largely from follow-up studies of children and adolescence with ADHD [e.g., Ref. (5, 6)]. It also seems that this co-morbid risk is exacerbated in girls (7), and that a common underlying bio-behavioral process influences both the risk for ADHD and for substance and alcohol dependence (8). In addition, in a recent long-term follow-up study, results were relatively consistent with most previous studies in finding that substance and alcohol abuse were about six times more likely in cases with ADHD than in controls, and that females had a significantly higher risk than males (9).

The ADHD-drug use/abuse link is also evident from the reverse perspective. It has been estimated that up to 50% of adolescents and adults with substance abuse disorders have a lifetime diagnosis of ADHD (5, 10). For example, ADHD was significantly more prevalent in methamphetamine abusers compared to control participants, especially in those with a persistence of symptoms into adulthood (11). Research also indicates that the comorbidity of substance use disorders and ADHD is associated with a more severe progression from use to abuse, and with greater social and psychiatric impairment (12).

In a recent 15-year, longitudinal, population-based ADHD study, the prevalence of substance abuse/dependence was substantially higher (≈31%), however, for nicotine than for other drugs like alcohol, cannabis, and cocaine (13). It also appears that the choice of addictive substance may be affected by the medication status of the user as seen in a case–control study of medication-compliant (i.e., methylphenidate or atomoxetine) adolescents. As anticipated, the ADHD probands were more likely than the controls to be daily smokers (14). However, contrary to expectation, the control participants reported heavier and more regular use of alcohol. A weaker link between ADHD symptoms and alcohol use, compared to that between ADHD and nicotine use, has also been found in other research (15). Such substance-related differences were explained, in part, by previous evidence that alcohol has a synergistic effect on methylphenidate by increasing its potency and causing feelings of dizziness and discomfort – effects that might discourage alcohol consumption in some individuals taking this medication (16).

### Medication status and risk for substance use/abuse

Stimulant medication for ADHD continues to be the first-line treatment for this disorder in most clinical settings, despite lingering concerns about its potential for abuse and whether it may sensitize individuals to later problematic substance use (17). Drugs like methylphenidate and amphetamines block or inhibit the dopamine and the norepinephrine transporters thereby increasing extracellular levels of these neurotransmitters. Amphetamines also gain access to the presynaptic terminals and foster the release of these catecholamines (18). As with all pharmacotherapies, however, efficacy of the drug varies across individuals and is influenced by brain neurochemistry and physiology. For instance, in two human studies, it was found that low levels of the dopamine D2 receptor in the striatum were associated with greater reinforcing responses to methylphenidate – a factor, which may predispose individuals with a hypo-functioning dopamine system to the risk of stimulant drug abuse (19, 20).

In an early meta-analysis of six stimulant-treatment outcome studies, Wilens and colleagues (21) concluded that pharmacotherapy for ADHD in childhood actually reduced the likelihood of later problem drug and alcohol use. However, a more rigorous review, of a larger body of empirical evidence a decade later, found no support for the “sensitization hypothesis” of stimulant treatment. Indeed, neither did it find that stimulant treatment conferred a protective effect on later substance abuse (17). Recent preclinical research suggests that inconsistencies in the putative relationship between stimulant treatment and risk for substance abuse may be explained by the moderating effects of emotional factors. For instance, juvenile rats chronically treated with methylphenidate showed a greater intake of, and preference for, alcohol in adulthood, but only in those who were socially isolated by being caged in solitary housing – an environment known to increase stress and anxiety in these animals (22).

Sex and age appear to be other factors that moderate the ADHD-drug use relationship. In a clinical population-based, birth-cohort study, it was found that childhood ADHD cases were 6.2 times more likely to have an alcohol/drug use disorder than non-ADHD controls from the same cohort, and that stimulant treatment tended to be a protective factor, but *only* in boys (23). In a more recent prospective arm of the same study, from the same birth cohort, it was confirmed that ADHD cases diagnosed in adolescence were more likely to have alcohol or drug dependence in adulthood (24). In other words, as ADHD cases grew to maturity, they were more likely to use drugs and were more likely to develop new-onset drug dependence than controls. Importantly, however, this study found that ADHD cases who had received treatment (for at least 6 months) after the age of 13 were at greater risk than those who received treatment before that age. Similarly, Dalsgaard et al. (9) found that both boys and girls with ADHD were at increased risk for substance abuse in adulthood, but that early initiation of stimulant treatment in children resulted in reduced risk compared to cases with later treatment onset. Nevertheless, there is still not complete agreement on the relationship between treatment with psychomotor stimulants and the risk for developing a drug addiction, nor the causal direction of such a putative association. Some of the outcome inconsistencies may be due to the relatively short length of follow-up and the high rates of attrition in earlier studies [e.g., Ref. (25, 26)].

### Mechanisms linking ADHD and substance use/abuse

Although links between ADHD symptomatology and substance (ab)use are well-documented, there has been little information about mechanisms that might foster this connection. One approach has been to examine the influence of personality traits associated with both ADHD and substance users in the general population. In this regard, the very limited ADHD research has focused largely on facets of impulsivity and their association with alcohol consumption in this clinical cohort [e.g., Ref. (27)]. Other research has indicated that the positive relationship between nicotine and marijuana use and ADHD-symptom dimensions may also be mediated by aspects of impulsivity (28). In addition, related investigations have found that an aversion to delayed gratification and an abnormal sensitivity to individual instances of reward are mediating links between symptoms of ADHD and addictive behaviors (29). These authors have suggested that a high reward drive might imply that “dopamine timing is off” in those with ADHD. Indeed, the pathophysiology of ADHD has mostly been ascribed to dopamine dysfunctions in the mesocorticolimbic pathway (30). Imaging studies have shown, for example, that ADHD patients display in increased availability of the dopamine transporter in this brain region relative to their healthy counterparts [see Ref. (31) for a review]. While there is other evidence that ADHD is associated with reduced functionality of the dopamine system – due in part to reduced receptor densities in various brain regions compared to non-affected individuals [see Ref. (32, 33)] – findings are not entirely consistent. For example, some studies suggest that ADHD is associated with a hyperactive dopamine system due either to an elevated efflux of dopamine or a reduced decrease in the reuptake of dopamine (34).

Results of recent longitudinal research have also shown that the development of internalizing problems such as depression and anxiety – largely through peer rejection – mediates the relationship between ADHD symptoms and risk for substance use and abuse (15). Indeed, several studies have reported that depression and anxiety disorders are the most frequently reported psychiatric co-morbidities in those with ADHD [see Ref. (35)]. Such data suggest that substance use, and other addictive behaviors, may be a form of “self-medication” in the absence of adequate social support, and as a means to cope with stressful events in adolescence. Together these studies mesh with evidence that high-risk profiles for substance misuse include anxiety sensitivity, impulsivity, and high sensation-seeking tendencies (36).

In the quest to better understand the mechanisms linking ADHD symptoms to addictive behaviors, no mediational research has examined whether stimulant-medication treatment for ADHD affects the hypothesized associations among the variables of interest. Moreover, the possible role of sex differences in moderating these associations is untested. To address these issues, the current study has employed a case-double-control design. Examining undiagnosed individuals with high-ADHD symptoms in the general community, as well as stimulant-treated clinical cases of ADHD, in relation to addictive behaviors removes the potential confounding effects of medication status on outcome.

### The current study

**Figure 1:**
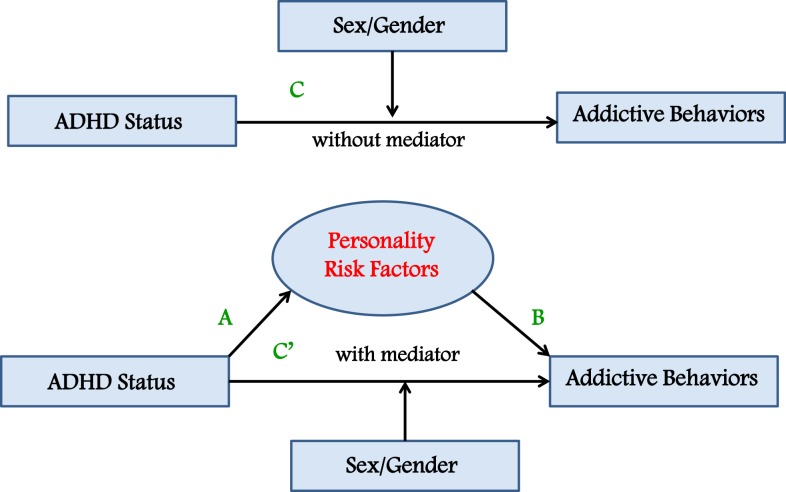
**Moderated-mediation model predicting that a personality-risk index mediates the relationship between ADHD status and a composite measure of addictive behaviors, and that sex moderates these associations**.

In this study, a moderated-mediation analysis was used to test our prediction that a composite index of personality risk – including impulsivity traits, reward sensitivity, and anxiety proneness – mediates the relationship between ADHD symptomatology on the one hand, and a general tendency toward engaging in addictive behaviors on the other. We also predicted that sex would moderate these relationships with males showing higher scores on all the measured variables compared to females (see Figure 1). These associations were assessed in three groups of young adult men and women: those with a previous or current diagnosis of ADHD who had been (or were currently being) treated with a stimulant medication (e.g., methylphenidate); a high-ADHD-symptom group; and, a low-ADHD-symptom group, both with no lifetime diagnosis of, or stimulant treatment for, ADHD. It was anticipated that the cases would have higher scores on all the measured variables in the analyses compared to the high-symptom control group, who, in turn, would have higher scores than the low-symptom controls.

## Materials and Methods

### Participants

A sample of young men (*n* = 98) and women (*n* = 116) between the ages of 17 and 32 years were recruited from the community of a large Canadian university (student enrollment is ≈55,000, with an additional 7000 faculty and staff employed on campus). Mean ages (and SD) of the participants were 22.5 (3.3) and 22.2 (3.3) years, for males and females, respectively. Among these individuals, 46 (men = 25; women = 21) had received a diagnosis of ADHD, and were either currently being treated with stimulant medications or had been in the past. The prescribed medications were Concerta, Ritalin, Vyvanse, Adderall, and Dexedrine. Participants were required to be fluent in written and spoken English and to have lived in North America since childhood. Exclusion criteria included a current diagnosis of an addiction disorder and a current or lifetime diagnosis of a psychotic disorder using an abbreviated (non-patient) version of the *structured clinical interview for DSM-IV* (SCID).

### Measures

#### ADHD status

ADHD status was established by participant self-report, each of whom was asked whether they had ever had a medical diagnosis of ADHD. If they answered in the affirmative, they were asked at what age the diagnosis took place and whether they were ever prescribed (and took) stimulant medication as part of their treatment protocol. If stimulants were taken, the participant was asked for the length and dates of the treatment, and for the name of the prescribed medication. Approximately half of the ADHD group was still on stimulant medication at the time of study participation, while the other half had a prior history of pharmacotherapy with stimulants, but was no longer receiving treatment at the time of recruitment. The non-diagnosed participants were dichotomized into a high-ADHD-symptom group (*n* = 83: females = 53) and a low-symptom group (*n* = 84: females = 41) based on a median split of their scores on the well-validated *Connors Adult ADHD Rating Scale* (CAARS) (37). The self-report measure was employed, which evaluates the presence and severity of ADHD symptoms. The scale comprises 30 items that are rated on a four-point scale based on the frequency and severity of ADHD inattentive and hyperactive/impulsive symptoms (0 = not at all, 1 = just a little, 2 = pretty much, and 3 = very much). In the present study, the total score was used and a median split of the data from the non-ADHD participants was used to define the low- and high-symptom groups.

#### Personality risk

Personality risk was modeled as a latent variable comprising three personality factors associated with impulsive and rash responding, and with anxiety proneness: (i) *Impulsivity* was assessed by the well-validated 30-item *Barratt Impulsivity Scale* (BIS) (38), which identifies facets of impulsivity such as the non-planning aspects of this construct, as well as the tendency to act rashly and to make quick decisions. The alpha coefficient in this study was 0.77; (ii) *Reward Sensitivity* was assessed by the Reward subscale (RS) of the *Sensitivity to Punishment and Sensitivity to Reward Questionnaire (SPSRQ)* (39). This scale comprises 24 forced-choice items reflecting the respondent’s approach responses under various conditions of reward. This scale was developed to assess the behavioral activation system (BAS) of Gray’s psychobiological model of personality (40, 41). The alpha coefficient for the present study was 0.78; and (iii) *Addictive Personality Traits* were assessed by the 32-item *Addiction Scale* (AS) of the *Eysenck Personality Questionnaire-Revised* (EPQ-R) (42). This scale was derived empirically by identifying those items of the EPQ-R, at or beyond the 0.001 level of significance – and irrespective of subscale – which differentiated male drug addicts from normal controls (43). In addition to studies with drug addicts (44), this scale has been validated with groups of problem drinkers (45), pathological gamblers (46), and those with anorexia nervosa, bulimia nervosa, and binge eating disorder (47, 48). The scale items are weighed toward the impulsive traits, as well as anxiousness and negative affect. The alpha coefficient in the present study was 0.81.

The three variables described above were moderately correlated, as expected (*r* between 0.33 and 0.41; all *p*-values <0.0001). A composite score was therefore calculated using principal component analysis, as described in the Section “Results.”

#### Addictive behaviors

Addictive behaviors were assessed by the *Shorter PROMIS Questionnaire* (49), a self-report instrument for the concurrent measurement of 16 addictive and/or excessive behaviors. Each subscale comprises 10 statements that the respondent endorses on a 6-point scale from 0 (“not like me”) to 5 (“like me”). The items for each scale reflect the common characteristics of addictive behaviors, such as use for effect, protection of supply, preoccupation, using more than intended and increased capacity or tolerance. For the purpose of the current study, a total score was created by summing the items for the following seven subscales: caffeine, recreational drugs, sex, nicotine, food binging, shopping/spending, and alcohol. Other subscales such as “compulsive helping – dominant/submissive” and “relationship – dominant/submissive” were deemed insufficiently related to conventional addiction disorders to be included in the aggregate score.

### Procedures

Participants were recruited by posters placed around the university campus, by newspaper advertisements, and by means of targeted announcements in online student forums. An initial screening took place during a short telephone interview. An appointment was made for a 1-h meeting in the university research laboratory of the first author for participants who appeared to meet the eligibility criteria. One the day of testing, informed consent and all relevant demographic and clinical information was obtained during a face-to-face interview. After the questionnaire package was completed, height and weight were measured with the participant standing in stocking feet and wearing light indoor clothing. At the completion of the study, each participant was paid $15.00 to cover out-of-pocket expenses. All study procedures were carried out according to the Declaration of Helsinki.

### Statistical analyses

The moderated-mediation model described in the Section “Introduction” was analyzed using the four-step procedures described by Baron and Kenny (50). According to this approach, mediation is present when the following conditions are met: (i) the independent categorical variable (ADHD status) is significantly related to the proposed mediator (personality risk), shown as path A in the model (see Figure 1); (ii) the proposed mediator (personality risk) is significantly related to the dependent variable (addictive behaviors), shown as path B in the model; (iii) the independent variable (ADHD status) is significantly related to the dependent variable (addictive behaviors), shown as path C in the model; and (iv) the relationship between ADHD status and addictive behaviors is substantially minimized – or becomes non-significant – when the proposed mediator (personality risk) is added as a covariate in the analysis of variance (ANOVA) analysis described in the third step. Sex was included as a potential moderator variable in the first, third, and fourth ANOVA analyses described above. Moderation is found if the ADHD status × sex interaction is statistically significant.

## Results

As a preliminary analysis, independent *t*-tests were used to assess group differences between the currently medicated and the previously medicated ADHD participants on all the quantitative variables used in this study. Since there were no significant differences between the groups, they were combined into a single group for all subsequent analyses.

Table 1 presents the means and SD for all quantitative variables included in the analyses, as well as for age and BMI, listed separately for the three ADHD-status groups (i.e., those with a diagnosis of ADHD, the high-symptom control group, and the low-symptom control group). The groups did not differ from each other on BMI. However, the low-symptom group was significantly older than the high-symptom (*p* = 0.033) and the ADHD (*p* = 0.008) groups, who did not differ from each other. Although statistically significant in our sample, an age difference of 1–2 years in young adulthood was not considered clinically relevant in the context of our research. With respect to the classification variable for the control groups (viz. symptom scores as assessed by the CAARS), the ADHD group had significantly higher scores than the low-symptom group (*p* < 0.0001). Surprisingly, there was no difference between the ADHD groups and the high-symptom control group (*p* = 0.456).

**Table 1:** **Means and SD for all quantitative variables listed separately for the three ADHD status groups**.

Variable	ADHD		High-symptom		Low-symptom	
	Mean	SD	Mean	SD	Mean	SD
Age	21.5	2.7	22.0	3.3	23.1	3.3
BMI	25.8	4.4	25.4	6.7	24.2	4.7
CAARS total score	41.4	20.6	40.0	11.3	15.3	6.4
Barrett impulsivity scale	71.3	14.9	67.3	11.5	55.7	7.8
Reward sensitivity	12.5	4.3	13.5	4.2	10.4	4.3
Addictive personality traits	13.3	6.2	14.5	4.7	9.8	4.4
Addictive behaviors	71.0	60.0	64.0	35.2	42.8	29.5

In order to create a latent variable reflecting personality risk, a composite score was calculated for the three personality variables (BIS, RS, and AS) using a principal component analysis. The extracted component accounted for 58% of the variance in the three personality scales, and all three loaded strongly on this factor (loadings ranged from 0.73 to 0.79).

### Model testing

#### Path A

Path A was tested using a 3 (ADHD status) × 2 (Sex) ANOVA with personality risk as the dependent variable. There was a significant main effect for ADHD status. *Post hoc* comparisons using the *least significant difference* (LSD) test indicated that the low-symptom group had significantly lower personality-risk scores than the ADHD group (*p* < 0.0001) and the high-symptom group (*p* < 0.0001), which did not differ from each other (*p* = 0.634). Neither the main effect for sex nor the AHDH status × sex interaction was statistically significant. Table 2 presents the summary statistics for these analyses.

**Table 2:** **Summary statistics for the 3 × 2 ANOVA with the personality-risk factor score as the dependent variable**.

Source	df	Mean squares	*F*	*p*-value
Intercept	1	0.7	0.89	0.346
ADHD status	2	23.7	31.80	**<0.0001**
Sex	1	0.3	0.45	0.502
ADHD status × sex	2	1.0	1.40	0.249
Error	203	0.7		
Total	209			

#### Path B

Path B was tested by regressing addictive behaviors on the personality-risk factor score, and results indicated a significant positive association between the two variables (see Table 3).

**Table 3:** **Unstandardized coefficients for the regression analysis with addictive behaviors as the dependent variable and the personality-risk factor score as the independent variable**.

Variable	*B*	SE	*t*(*H*_o_)	*p*
Intercept	57.4	2.3	25.11	**<0.0001**
Personality risk	25.5	2.3	11.14	**<0.0001**

**R* ^2^ = 0.38*.

#### Path C

Path C (without the mediating variable) was tested using a 3 (ADHD status) × 2 (sex) ANOVA with addictive behaviors as the dependent variable. There was a significant main effect for ADHD status. *Post hoc* comparisons using the LSD test again indicated that the low-symptom group had significantly lower scores on the addictive-behaviors variable than the ADHD group (*p* < 0.0001) and the high-symptom group (*p* = 0.001), which did not differ from each other (*p* = 0.348). Neither the main effect for sex nor the AHDH status × sex interaction was statistically significant. Table 4 presents the summary statistics for these analyses.

**Table 4:** **Summary statistics for the 3 × 2 ANOVA with the addictive behaviors as the dependent variable**.

Source	df	Mean squares	*F*	*p*-value
Intercept	1	664358.0	418.34	<0.0001
ADHD status	2	13642.9	8.59	**<0.0001**
Sex	1	2292.1	1.44	0.231
ADHD status × sex	2	1073.1	0.68	0.510
Error	206	1588.1		
Total	212			

#### Path C′

In the final step, *Path C′* was tested by repeating the analysis described in Section “Path C”; however, this time the proposed mediator (personality risk) was included as a covariate in the model. Results indicated that personality risk was a highly significant predictor in the model, but that the ADHD status main effect no longer contributed significantly to the variance in addictive behaviors. There was no main effect for sex, nor was the ADHD status × sex interaction statistically significant (Table 5).

**Table 5:** **Summary statistics for the 3 × 2 ANOVA with addictive behaviors as the dependent variable and the personality-risk factor score as a covariate in the model**.

Source	df	Mean squares	*F*	*p*-value
Intercept	1	614323.1	569.3	<0.0001
Personality risk	1	107627.9	99.34	**<0.0001**
ADHD status	2	1352.7	1.2	0.288
Sex	1	984.3	0.91	0.341
ADHD status × sex	2	2571.5	2.38	0.095
Error	201	1079.12		
Total	208			

### Supplementary analyses

Although we selected an aggregate index in this study to reflect a *general* proclivity for addictive behaviors, we also expect that some readers may be interested in the statistical outcome for each individual addictive behavior. The following provides a summary of these results, and should be viewed as information supplementary to the original hypothesis-driven analyses. Table 6 presents the means and SD for each of the seven addictive behaviors, listed separately for the ADHD status groups and for men and women. To assess group differences, we employed a 3 (ADHD status) by 2 (sex) multivariate analysis of variance (MANOVA) with the individual addictive variables as dependent variables. The multivariable Wilks’ Lambda *F* ratios were statistical significant for ADHD status and for sex (*F*_14,400_ = 3.33; *p* < 0.0001, and *F*_7,200_ = 7.12; *p* < 0.0001, respectively). However, the main-effects interaction term did not reach statistical significance. Results of the univariate results are provided below.

**Table 6:** **Means and SD for seven addictive-behaviors subscales of the PROMIS questionnaire, listed separately for the three ADHD status groups and for sex**.

Variable	ADHD		High-symptom		Low-symptom	
	Mean	SD	Mean	SD	Mean	SD
**Female**
Caffeine	3.9	5.1	3.9	4.6	1.5	2.8
Recreational drugs	12.9	18.5	3.3	7.1	2.3	5.6
Sexual activities	7.2	13.9	3.8	8.6	2.3	6.1
Nicotine	8.6	14.6	2.6	8.4	1.9	6.1
Food binging	13.2	13.6	16.0	9.8	12.3	6.1
Alcohol	9.0	10.4	9.7	10.7	8.3	9.3
Shopping/spending	13.9	12.5	17.0	9.9	13.6	9.4
**Male**
Caffeine	4.7	7.8	5.9	7.4	4.9	6.9
Recreational drugs	12.4	15.4	7.2	10.7	3.8	7.4
Sexual activities	10.3	13.7	9.9	9.5	6.4	8.2
Nicotine	8.2	15.4	6.6	11.7	2.1	6.2
Food binging	10.0	10.0	14.9	8.1	6.8	6.1
Alcohol	15.4	11.4	15.4	11.0	11.2	8.8
Shopping/spending	10.8	9.8	11.5	8.6	8.3	7.2

#### Caffeine intake, alcohol consumption, and shopping/spending

For these three variables, there were no main effects for ADHD status. However, males had a higher frequency of caffeine (*p* = 0.01) and alcohol (*p* = 0.01) consumption compared to females, while females were more prone to compulsive shopping (*p* = 0.001).

#### Recreational drugs and nicotine

In contrast, for these two addictive behaviors there were no main effects for sex. There were, however, significant main effects for ADHD status (*p* < 0.0001 and 0.002, respectively). *Post hoc* comparisons using the LSD procedure indicated that for recreation drug use, the ADHD group had significantly higher scores than both the low- and the high-symptom groups (*p* < 0.0001 in each case), who did not differ significantly from each other. With respect to nicotine, similar results were found with the ADHD group having higher scores than the high-symptom (*p* = 0.026) and the low-symptom (*p* < 0.0001) groups, who again were not different from each other.

#### Food binging and sexual activity

For these two variables, there was a significant additive effect of ADHD status (*p* = 0.001 and 0.045, respectively) and sex (*p* = 0.025 and 0.002, respectively). Not surprisingly, woman had higher scores on food binging than men, while the reverse was found for sexual activity. Concerning food binging, the high-symptom group had more elevated scores than either the low-symptom (*p* = 0.001) or the ADHD groups (*p* = 0.027), who did not differ from each other. And finally, the low-symptom group had reduced scores on the sexual-activity variable compared to the high-symptom (*p* = 0.037) and the ADHD (*p* = 0.004) groups, who were not significantly different from each other.

## Discussion

Results of our moderated-mediation analysis suggest that a composite index of personality risk – reflecting aspects of impulsivity and reward drive, as well as neurotic and anxiousness traits – may mediate the positive relationship between ADHD symptomatology and addictive behaviors. In other words, these findings suggest that the personality traits frequently found in those with ADHD may be the underlying mechanism driving their preference for, and proneness to engage in, activities with immediately reinforcing qualities and outcomes. Unexpectedly, however, there were no differences between the ADHD group and the high-symptom participants on the composite measure of addictive behaviors, although both groups had significantly higher scores than the low-symptom group^1^. We also found that the ADHD and the high-symptom group had virtually identical scores on the ADHD-symptom variable (viz. the CAARS), which is used clinically as a diagnostic tool, and was employed in this study to dichotomize the sample into high- and low-symptom control groups. The findings described here suggest that stimulant medication (either current or past) does not appear to enhance or diminish the general likelihood of engaging in addictive activities. In essence, these results appear to be in accord with recent research showing no evidence of a “sensitization” effect of stimulant treatment in those with a lifetime diagnosis of ADHD (17). Such conclusions, however, must be interpreted with caution since the sample of participants with ADHD was not of sufficient size to control for factors such as length of stimulant treatment, age of onset, and medication dosage.

The absence of clinically relevant symptom differences between the ADHD and the high-symptoms groups may suggest that the former was more high-functioning than is typical of the general population of young adults with ADHD (or a history thereof). Indeed, this possibility gains credibility since most of the ADHD participants in our study were recruited from the student body of a local university. On the other hand, a recent survey of childhood impairments in those with and without ADHD – based on retrospective adult recall – found that while the ADHD group reported more school difficulties compared to controls, there were no differences in their respective levels of educational attainment (35). Such data imply, therefore, that university recruitment does not necessarily create a biased sample in relation to the severity of ADHD symptomatology. Another possible explanation for the absence of differences between the ADHD and the high-symptom groups is that stimulant treatment for the former may have ameliorated their symptom severity. On the other hand, the absence of differences may also indicate that ADHD is relatively under-diagnosed among apparently healthy adults who have high-ADHD symptoms.

It was of considerable interest to find no differences in the magnitude of the personality-risk index between those with AHDH and the high-symptom group, although again both had significantly higher scores than the low-symptom group. The composite personality variable – created for the current study as a marker of risk for addictive behaviors – is not only validated empirically by our results but also complements previous research in this area. For instance, both novelty seeking and harm avoidance were significantly greater in a large group of patients with opiate addiction and/or alcohol dependence compared to normal controls (52). Individuals with a compulsive buying disorder also had more symptoms of ADHD, as well as lifetime mood, anxiety, and impulse control disorders, compared to an appropriate control group (53). Additionally, the compulsive buyers had more pronounced personality traits related to depression, impulsivity, and novelty seeking (an aspect of high-reward sensitivity).

Contrary to expectations, and after controlling for ADHD status in our analyses, there were no male–female differences in personality risk, nor in the use of addictive behaviors; neither did sex moderate the relationships tested in our mediation model. Concerning the addictive-behaviors variable – and in light of our findings that individual differences in risk were not sex-specific – it may be that sex differences were washed out because we operationalized addictive behaviors as an aggregate index of several activities, both substance and non-substance related. Some addictive behaviors, like compulsive buying (54) and binge eating (55), are more frequently found in women, while others like hypersexual behaviors tend to be more common in men (56)^2^.

In conclusion, strengths of this study are the inclusion of a non-diagnosed and non-treated group of young adults with high-ADHD symptom severity equivalent to the group of clinically diagnosed ADHD participants. In addition, we also included a low-symptom control group, thereby providing a double control for the clinical cases. Our focus on both mediating and moderating factors in connection to the ADHD-addictive behaviors link is another strong point of this research. The use of a composite dependent-variable index of addictive behaviors also provided a more comprehensive approach than one which examined each addictive activity separately (although these data have been provided as supplementary information). This strategy is particularly relevant since preferences for specific addictive activities are known to vary across sociocultural groups (59–61). In addition, while other studies have examined personality correlates of ADHD – in particular, those also associated with risk for addiction – as reviewed in the Section “Introduction,” this body of work has largely investigated constructs related to impulsive responding. The current study has extended this research by using a multivariate approach to operationalize personality risk by forming of composite latent variable including facets of impulsivity, reward sensitivity, and anxiousness. We have also moved beyond the investigation of simple relationships by employing moderated-mediation procedures in our data analyses.

However, despite the merits of our current research, it is also important to address the limitations of the study. Foremost is the fact that the ADHD participants comprised two distinct sub-groups – those who were currently on stimulant medication and those who had been, but were no longer, taking these drugs. While our data indicated that the two groups did not differ on the variables included in this study, these analyses may have been under-powered by virtue of relatively small sample sizes. Another constraint of the study is that the associations we observed were based on cross-sectional data, thereby limiting our ability to infer directional relationships between symptoms and behaviors. While it is intuitive, for example, to suppose that ADHD symptoms contribute to the use and abuse of addictive behaviors, it is also known that chronic use of addictive substances/activities can foster some of the symptoms that define ADHD such as poor impulse control (62). Only longitudinal research will be able to establish causal mechanisms between ADHD symptoms and addiction, and the mediating role of personality-risk factors.

To summarize, we found that a high-risk personality profile may, in part, account for the relationship between ADHD symptomatology and the use/abuse of a broad range of addictive behaviors. We also found no evidence that current or past treatment of ADHD symptoms with stimulant medication increases the probability of engaging in potentially addictive activities. While there is good evidence that ADHD is more prevalent in males than in females (63), we found no sex differences in personality risk for addiction or in the use of addictive behaviors; nor did sex moderate the relationships we assessed. The mediational impact of personality-risk factors found in our study has important clinical implications, especially in light of recent evidence from a randomized control trial, demonstrating that a personality-targeted prevention program for adolescence was significantly more effective in reducing alcohol use and misuse than a standard and statutory drug-education program (36).

## Conflict of Interest Statement

The authors declare that the research was conducted in the absence of any commercial or financial relationships that could be construed as a potential conflict of interest.

## Footnotes

^1^When each addictive behavior was considered separately – as seen in the Section “Supplementary Analyses” – recreational drug use and cigarette smoking were significantly higher in the ADHD group than in either of the control groups, who did not differ from each other. Moreover, there were no group differences regarding alcohol consumption. It is interesting to note that the nicotine and alcohol results in the current study are in close accord with previous findings reported in the Section “Introduction” (14, 15). With regard to food binging, the finding that the high-symptom group had significantly elevated scores, compared to the ADHD or low-symptoms groups, is difficult to explain. Given that half of the ADHD group was currently on stimulant medication, and in light of recent evidence that stimulants are effective in reducing binge-eating episodes (51), it may be that the relatively low scores in the ADHD group were the result of medication effects. Importantly, however, and similar to results with the addictive-behaviors composite score, there was no ADHD status × sex interaction for any of the individual variables.

^2^Indeed, results from the *post hoc* Supplementary Analyses did demonstrate that women had higher scores than men on the binge-eating and compulsive-shopping variables, as seen in previous research (54, 55). We also found, as others have, that men had higher scores than women on sexual addiction (56) and alcohol consumption (57). With respect to caffeine, our findings showed that men also had higher scores than women. While very few studies have examined sex/gender differences in caffeine consumption, one early report indicated that adolescent boys found the reinforcing effects of caffeine greater than girls did, suggesting that the former may be more prone to consume caffeinated beverages (58). Important, however, is that sex did not moderate the relationship between ADHD status and addictive behaviors whether we used the composite index or the individual sub-scale scores.
